# Effects of Sodium Butyrate and Its Synthetic Amide Derivative on Liver Inflammation and Glucose Tolerance in an Animal Model of Steatosis Induced by High Fat Diet

**DOI:** 10.1371/journal.pone.0068626

**Published:** 2013-07-05

**Authors:** Giuseppina Mattace Raso, Raffaele Simeoli, Roberto Russo, Anna Iacono, Anna Santoro, Orlando Paciello, Maria Carmela Ferrante, Roberto Berni Canani, Antonio Calignano, Rosaria Meli

**Affiliations:** 1 Department of Pharmacy, University of Naples “Federico II”, Naples, Italy; 2 Department of Veterinary Medicine and Animal Productions, University of Naples “Federico II”, Naples, Italy; 3 Department of Translational Medicine–Pediatric Section and European Laboratory for the Investigation of Food Induced Diseases, University of Naples “Federico II”, Naples, Italy; Bambino Gesu' Children Hospital, Italy

## Abstract

**Background & Aims:**

Nonalcoholic fatty liver disease (NAFLD) is the most common form of chronic liver disease. Insulin resistance (IR) appears to be critical in its pathogenesis. We evaluated the effects of sodium butyrate (butyrate) and its synthetic derivative N-(1-carbamoyl-2-phenyl-ethyl) butyramide (FBA) in a rat model of insulin resistance and steatosis induced by high-fat diet (HFD).

**Methods:**

After weaning, young male Sprague-Dawley rats were divided into 4 groups receiving different diets for 6 weeks: 1. control group (standard diet); 2. HFD; 3. HFD plus butyrate (20 mg/kg/die) and 4. HFD plus FBA (42.5 mg/Kg/die, the equimolecular dose of butyrate). Liver tissues of the rats were analyzed by Western blot and real-time PCR. Insulin resistance, liver inflammation and Toll-like pattern modifications were determined.

**Results:**

Evaluation of these two preparations of butyrate showed a reduction of liver steatosis and inflammation in HFD fed animals. The compounds showed a similar potency in the normalisation of several variables, such as transaminases, homeostasis model assessment for insulin resistance index, and glucose tolerance. Both treatments significantly reduced hepatic TNF-α expression and restored GLUTs and PPARs, either in liver or adipose tissue. Finally, FBA showed a higher potency in reducing pro-inflammatory parameters in the liver, via suppression of Toll-like receptors and NF-κB activation.

**Conclusions:**

Our results demonstrated a protective effect of butyrate in limiting molecular events underlying the onset of IR and NAFLD, suggesting a potential clinical relevance for this substance. In particular, its derivative, FBA, could represent an alternative therapeutic option to sodium butyrate, sharing a comparable efficacy, but a better palatability and compliance.

## Introduction

A close association between non-alcoholic fatty liver disease (NAFLD) and several findings indicative of insulin resistance (IR) and metabolic disorders has long been reported. The liver produces and is exposed to various types of lipids, such as fatty acids, cholesterol and triglycerides via the portal vein from the diet and visceral adipose tissues. The liver and adipose tissue jointly participate in maintaining glucose and lipid homeostasis through the secretion of several humoral factors and/or neural networks. Perturbation in the inter-tissue communications may be involved in the development of IR and diabetes. An excessive free fatty acids (FFAs) flux into the liver via the portal vein may cause fatty liver disease and hepatic IR. However, the initial events triggering the development of IR and its causal relations with dysregulation of glucose and fatty acids metabolism remain unclear.

It has been suggested that the blood glucose- and lipid-lowering effects of soluble dietary fibres may be related in part to short chain fatty acids (SCFAs) generated during anaerobic microbial fermentation [1,2]. Among SCFAs, butyrate constitutes one of the major products derived from intestinal fermentation of undigested dietary carbohydrates, specifically resistant starches and dietary fibres, but also in a minor part by dietary and endogenous proteins. After butyrate uptake by the colon, it is metabolized in part by the colonocytes, and the remaining fraction reaches the liver via the portal vein. The colonocytes absorb butyrate through different mechanisms of apical membrane uptake, including non-ionic diffusion, SCFA/HCO_3_^-^ exchange, and active transport by monocarboxylic acid transporter (i.e. MCT1) [3]. In particular, butyrate is able to exert a powerful pro-absorptive stimulus on intestinal NaCl transport and an anti-secretory effect towards Cl^-^ secretion [4,5].

The effects exerted by butyrate are multiple and involve several distinct mechanisms of action. Its well-known epigenetic mechanism, is the hyperacetylation of histones by inhibiting class I and class II histone deacetylases (HDAC), that results in the regulation of gene expression and in the control of cell fate [6,7]. HDAC regulates gene transcription through modification of chromatin structure by acetylation of proteins, including not only histone proteins, but also transcription factors (i.e. NF-κB, p53 and NFAT) [8]. Butyrate also acts as signal molecules, targeting their G protein-coupled receptors Free Fatty Acid Receptor 2 (FFAR2, GPR43) and FFAR3 (GPR41) [9]. At intestinal level, butyrate exerts multiple effects, such as the prevention and inhibition of colonic carcinogenesis, the improvement of inflammation, oxidative status, epithelial defense barrier, and the modulation of visceral sensitivity and intestinal motility. At the extraintestinal level, potential fields of application for butyrate seem to be the treatment of different pathologies, including metabolic diseases, such as hypercholesterolemia, obesity, IR, and ischemic stroke [10].

Recently, it has been demonstrated that dietary supplementation of butyrate can prevent or treat diet-induced IR in mice [11]. The mechanism of butyrate action was related to promotion of energy expenditure and induction of mitochondrial function through stimulation of peroxisome proliferator activated-receptor (PPAR) γ coactivator (PGC)-1α. Moreover, activation of AMP kinase (AMPK) and inhibition of HDAC could contribute to PGC-1α regulation by butyrate. More recently Li et al [12] demonstrated that fibrobloblast growth factor (FGF) 21, which plays an important role in lipid metabolism [13], is induced by butrate and involved in the stimulation of fatty acid β-oxidation in liver.

Some butyrate-based products are marketed but their spread is still very limited and greatly understaffed in view of the wide spectrum of possible indications especially in chronic diseases where it is possible to predict a lasting use of these compounds. The unpleasant taste and odour make extremely difficult the oral administration of butyrate, these difficulties are even more remarkable in children where the administration is complicated. Thus, new formulations of butyrate with a better palatability, which can be easily administered orally, are needed. The purpose of this study is to investigate the efficacy of sodium butyrate (butyrate) and of its more palatable derivative, the N-(1-carbamoyl-2-phenyl-ethyl) butiramide (FBA), in a rat model of NAFLD induced by high fat diet. We hypothesized that orally administered butyrate compounds, could attenuate steatosis and liver injury, with reduction of inflammatory responses via suppression of Toll-like receptors (TLRs) through downregulation of NF-κB activation**.**

## Results

### Effects of butyrate and FBA on liver steatosis and serum parameters

**Figure 1:**
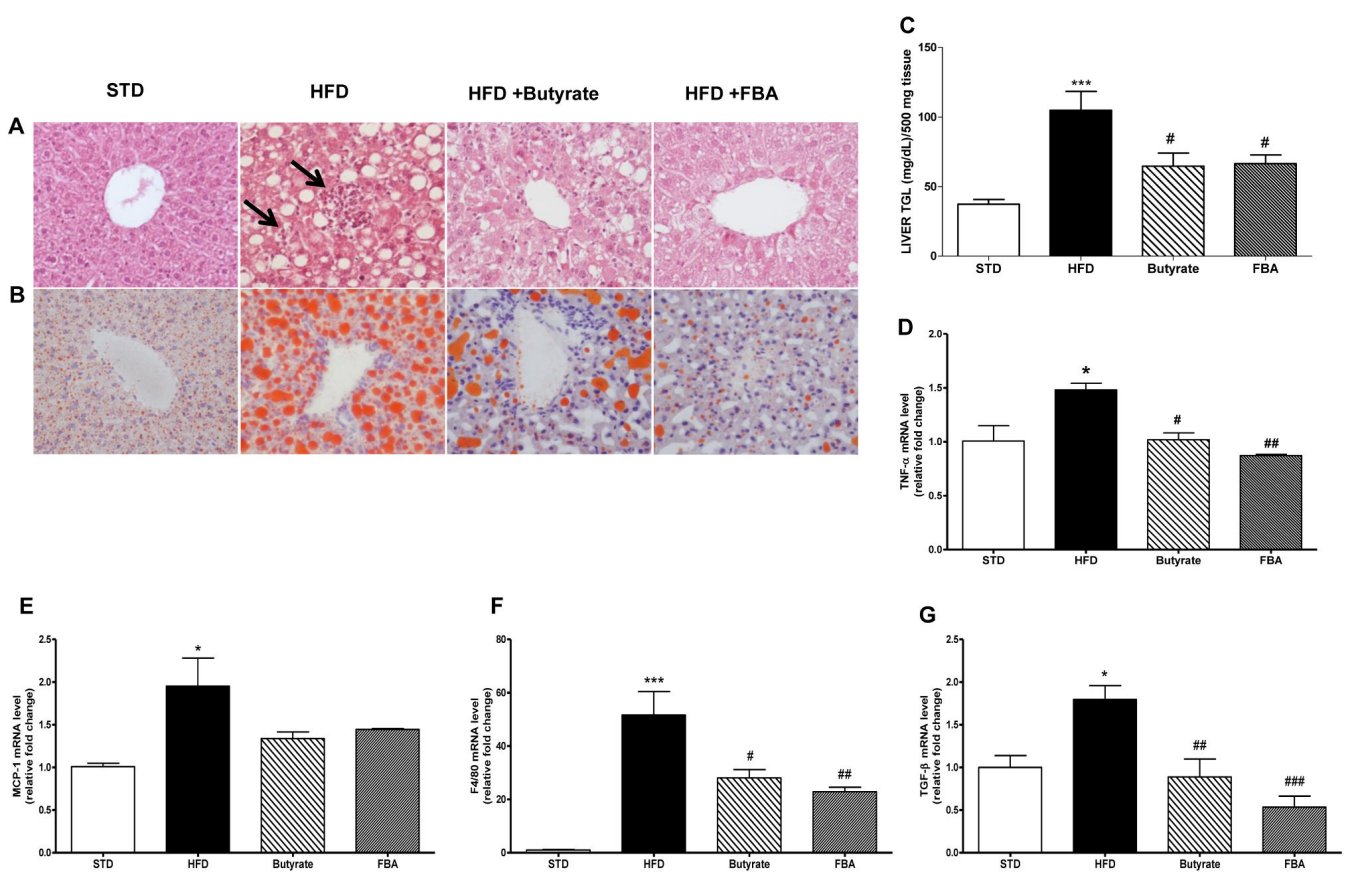
Effects of butyrate and FBA on liver steatosis. Paraffin-embedded sections of the liver (n=4 each group) were stained with hematoxylin-eosin (A) or oil red O (B). Micrographs in both panels are representative pictures with original magnification 40X. Foci of inflammatory cells are shown (arrows). (C) Triglycerides were measured in liver and normalized on 500 mg of frozen tissue (n=6 each group). The mRNA levels of pro-inflammatory cytokines TNF-α (D), MCP-1 (E), F4/80 (F), and TGF-β (G) were analyzed by real-time PCR in liver extracts (n=6, each group). *P<0.05, ***P<0.001 *vs* STD; ^#^ P<0.05, ^# #^ P<0.01, ^# # #^ P<0.001 *vs* HFD.

Liver sections from rats fed with HFD demonstrated significant hepatic damage in comparison with standard diet (STD) fed animals. As depicted in Figure 1 A, HFD livers showed foci of mixed inflammatory cell infiltration, evidenced by arrows, and hepatocyte necrosis or apoptosis throughout the lobule. Scattered inflammation and occasionally apoptotic nuclei were observed. No alterations were shown in liver of the rats fed with the STD. Furthermore, HDF rats showed grade 3 hepatic steatosis with a histological pattern characterized by microvesicular steatosis. The hepatocytes showed the cytoplasm filled with small vacuoles which were uniform in size and smaller than the centrally located nucleous. Steatosis affected most of the hepatocytes (Figure 1 B). In animals treated with equimolecular doses of butyrate (sodium butyrate or FBA), liver inflammatory damage appears reduced and steatosis was graded as grade 1 with a microvesicular pattern of lipid accumulation distributed in perivenular and periportal region. This effect was also associated with a reduction of triglycerides content which was significantly enhanced by HFD (Figure 1 C).

In HFD-fed animals TNF-α, a cytokine involved in the development of IR, was significantly increased, butyrate and in particular FBA reduced hepatic expression of this cytokines (Figure 1 D). Similarly the mRNA concentration of chemokine MCP-1 and a specific marker of mature macrophage f4/80 were increased in HFD rats and normalized by butyrate treatments (Figure E and F). Moreover, HFD induced the expression TGF-β1, an early marker of the fibrotic process (Figure 1 G), which was significantly down-regulated in the butyrate-treated group (P < 0.01) and more significantly by FBA (P < 0.001). We also evaluated α-SMA and pro-collagen type 1 transcript by real time-PCR but it did not show a significant modification after 6 weeks of HFD. Biochemical serum parameters are reported in table 1. Circulating levels of AST, ALT and cholesterol resulted increased at 6 weeks of HFD, while LDL and triglycerides showed a trend of increase. All these parameters were reduced by butyrate and FBA. Compared with animals fed with STD diet, HFD rats showed a marked increase in fasting glucose without change in insulin levels. Both butyrate and FBA prevented glucose alteration. Homeostasis model assessment for insulin resistance (HOMA-IR) was 25% and 30% lower in butyrate and FBA groups, respectively.

**Table 1:** Changes in serum parameters of rats fed with control standard diet (STD), high fat diet (HFD) or high fat diet and treated with Butyrate or FBA for 6 weeks.

	**STD**	**HFD**	**HFD+Butyrate**	**HFD+FBA**
ALT (U/l)	26.17±3.40	38.83±3.69*	22.00±1.59^##^	18.83±1.56^###^
AST (U/l)	157.33±12.23	226.16±24.15**	152.0±3.65^##^	146.60±12.79^##^
Colesterol (mg/dl)	71.50±1.72	86.14±6.37*	74.50±1.52^#^	74.67±3.03^#^
LDL (mg/dl)	24.55±0.74	27.23±2.39	20.40±0.86^#^	23.46±0.82
TGL (mg/dl)	38.50±3.52	43.86±3.70	35.17±0.40	39.67±1.62
Glucose (mg/dl)	110.3±3.22	154.0±16.27*	115.2±8.22^#^	118.4±5.48^#^
Insulin (µg/l)	0.20±0.06	0.27± 0.02	0.21±0.01	0.20±0.01
HOMA index	0.97±0.03	1.56±0.16**	1.17±0.11^#^	1.13±0.06^#^

Values are means ± S.E of six animals. *P<0.05, **P<0.01 vs STD. #P<0.05; ##P<0.01; ###P<0.001 vs HFD.

No difference in body weight was shown among all groups, weight gain of HFD fed animals did not change after 6 weeks neither modified by butyrate and FBA treatments (data not shown).

### Modulation of hepatic inflammatory parameters and NF-κB activation by butyrate and FBA

**Figure 2:**
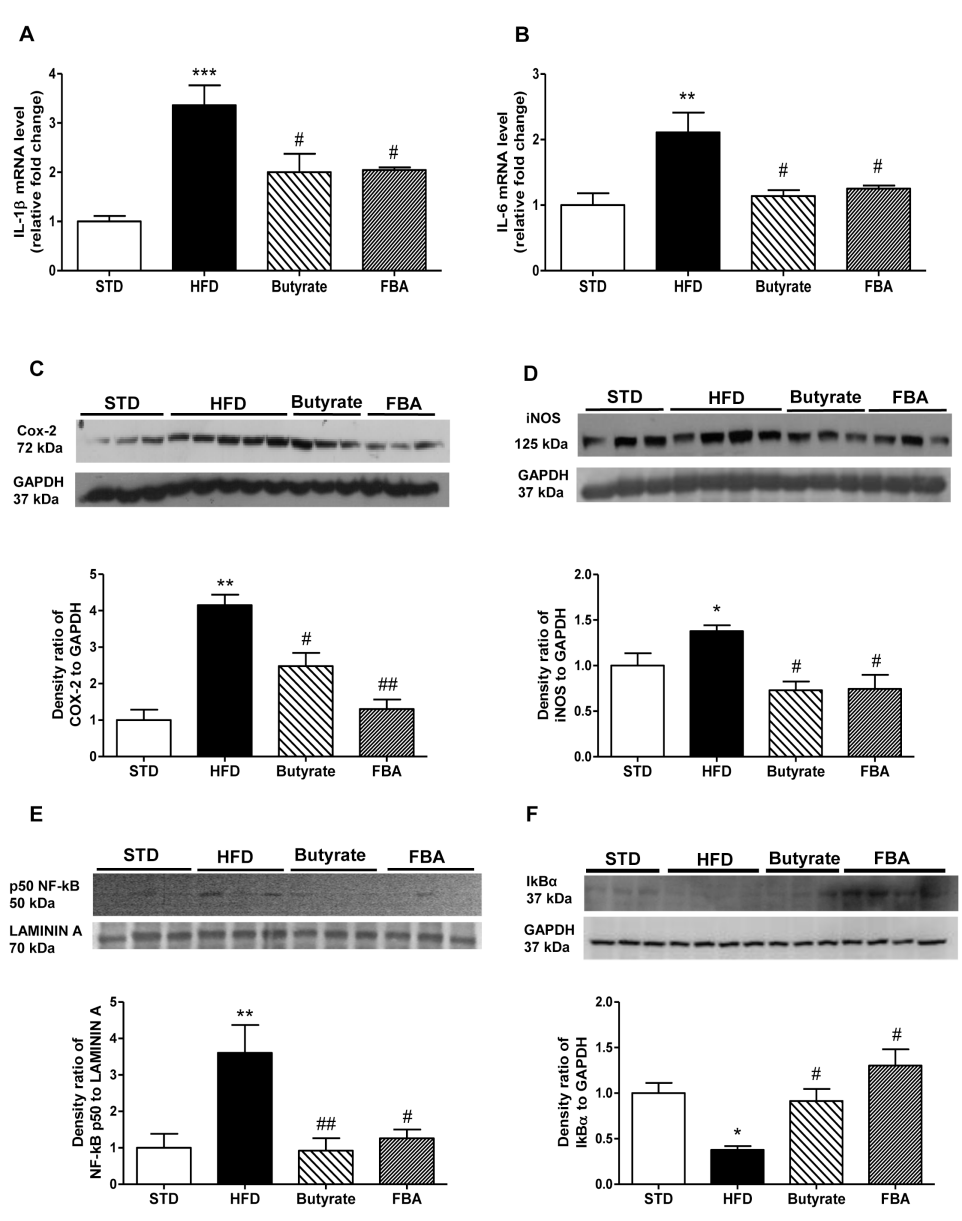
Modulation of hepatic inflammatory parameters and NF-κB activation by butyrate and FBA. IL-1β (A) and IL-6 (B) mRNA expression (relative fold change to STD) are reported (n=6, each group). Panels C and D show representative Western blot analysis of pro-inflammatory enzymes COX-2 and iNOS, respectively, in the liver extracts. Nuclear p50 NF-κB (E), and cytosolic inhibitory protein IκB-α (F) expression is reported (n=6 each group). *P<0.05, **P<0.01, ***P<0.001 *vs* STD; ^#^ P<0.05, ^# #^ P<0.01 *vs* HFD.

Hepatic and extra-hepatic IL-1 sources contribute to liver inflammation related to metabolic alterations. In fact, IL-1β is strongly up-regulated by activated macrophages or other liver cell types (including Kupffer cells and hepatic stellate cells), participating in liver injury. Beyond TNF-α, also IL-6 is involved in metabolic impairment, initiating the pathogenesis of hepatic IR. As shown in Figure 2 A and B, HFD induced a significant increase in hepatic IL-1β and IL-6 mRNAs, and both treatments significantly prevented the transcription of these genes. Moreover, the high-fat diet determined an increase in liver pro-inflammatory enzymes, COX-2 and iNOS (Figure 2 C and D) and butyrate and FBA reduced the expression of these proteins. In agreement both treatments significantly prevented the increase of nuclear content of p50 NF-κB related to a reduction of the inhibitory cytosolic protein IκBα, showing the inhibition of NF-κB activation (Figure 2 E and F).

### Effect of butyrate and FBA on hepatic Toll-like receptors pattern

**Figure 3:**
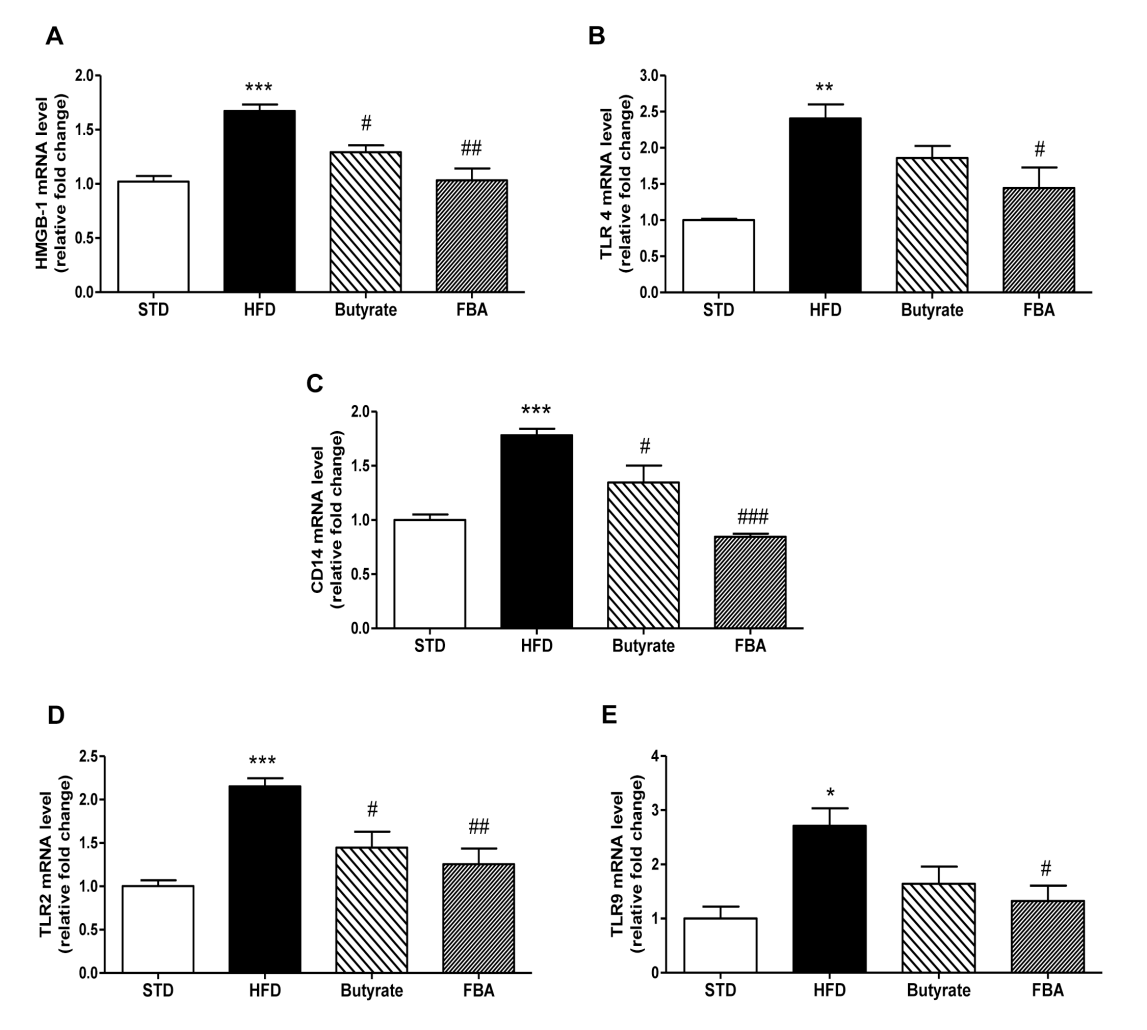
Effect of butyrate and FBA on hepatic Toll-like receptors pattern. Panels A and B are the results of real-time PCR for HMGB-1 and TLR4, respectively, in liver extracts from 6 rats on STD, HFD, HFD+butyrate, and HFD+FBA. Panel C shows mRNA expression of coreceptor CD14. TLR2 (D) and TLR9 (E) mRNA levels are shown and expressed as relative fold change to STD. *P<0.05, **P<0.01 and ***P<0.001 *vs* STD; ^#^ P<0.05, ^# #^ P<0.01, ^# # #^ P<0.001 *vs* HFD.

The activation of TLRs family, especially TLR4, by inflammatory cytokines or increased NEFA could modulate insulin sensitivity [14]. Recently, it has been hypothesized that FFA-related high–mobility group box 1 (HMGB1) release mediates the activation of TLR4 signaling in hepatocytes and plays an essential part in the early stage of NAFLD induced by HFD [15]. Here, HMGB-1 mRNAs were strongly up-regulated by HFD feeding while both butyrate, and in particular FBA, determined a significant reduction of these levels (Figure 3 A).

As shown in Figure 3B and C, HFD induced the mRNA increase of TLR4 and its co-receptor CD14 in liver. FBA and, to a lesser extent, butyrate inhibited these effects. Notably, TLR2 and TLR9, which are able to detect lipoproteins and unmetilated CpG-containing DNA, respectively, were also up-regulated by HFD and reduced by two treatments, with a great effectiveness of the FBA (Figure 3D and E).

### Effect of butyrate and FBA on glucose homeostasis and insulin resistance

**Figure 4:**
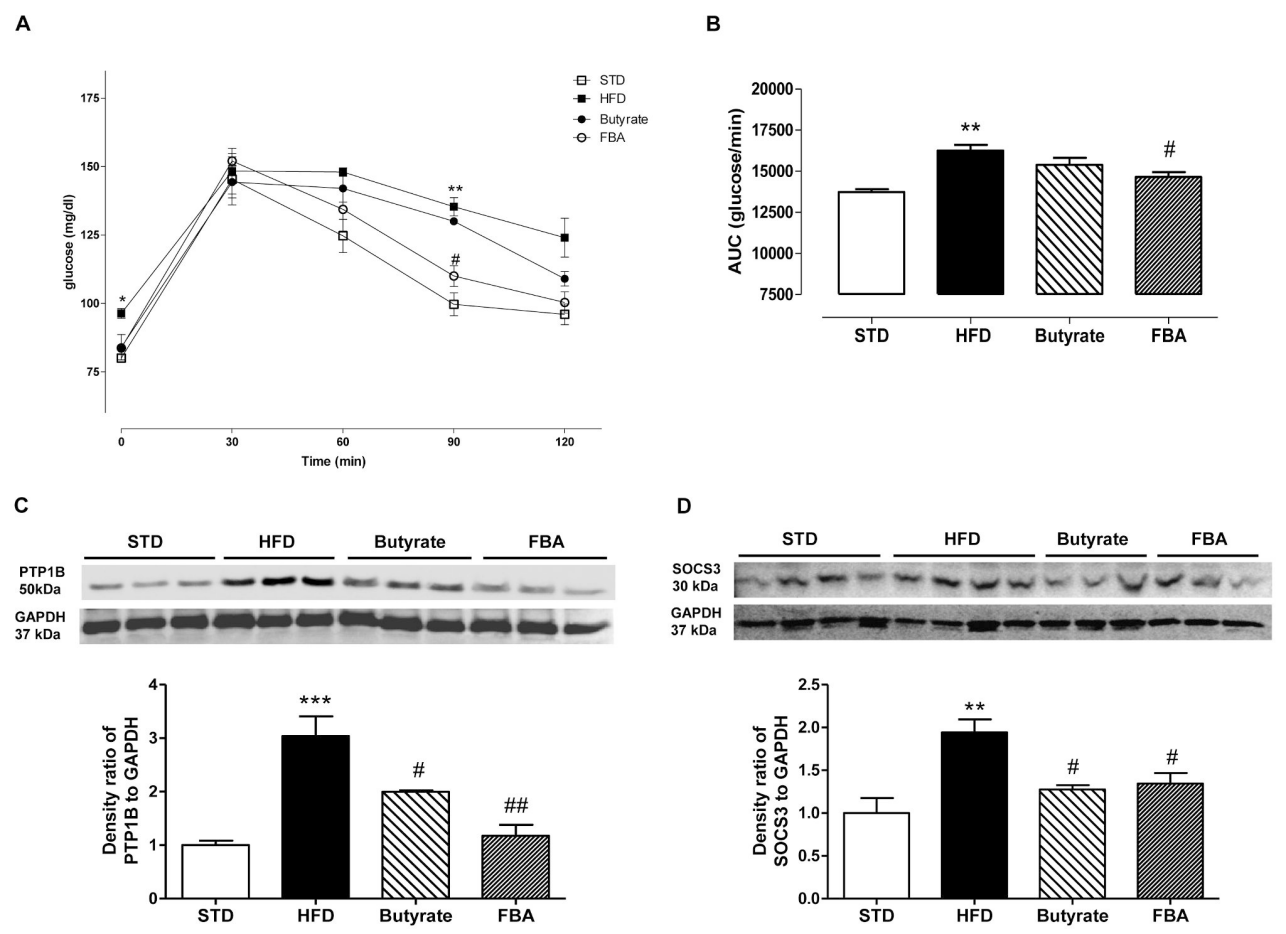
Effect of butyrate and FBA on glucose homeostasis and insulin resistance. Glucose tolerance test (A) in STD and HFD-fed rats (n=6, each group) was performed and AUC evaluated (B). Panels C and D show representative Western blot analysis of PTP1B and SOCS3 expression from liver tissue, respectively (n=6 each group). **P<0.01, ***P<0.001 *vs* STD; ^#^ P<0.05, ^# #^ P<0.01*vs* HFD.

To confirm the effects of butyrate and its derivative on insulin resistance in HFD-fed rats, glucose tolerance and insulin signaling were analyzed. In oral glucose tolerance test, blood samples were collected sequentially before, and 30, 60, 90, and 120 min after the glucose load, HFD induced an altered response to glucose at all time points paralleled with ST (Figure 4 A and B): in fact, a marked and significant increase of AUC values was shown in HFD group (P<0.001), which was significantly reduced by FBA (Figure 4 A and B). Butyrate and FBA groups exhibited improved response to glucose at all time points, in particular FBA raised a significant reduction of glycemia 90 min after glucose load (Figure 4 A).

The insulin receptor is acted on an unique group of regulatory proteins, including protein tyrosine phosphatase 1B (PTP1B), which is considered as a major negative regulator of insulin receptor signaling. As depicted in Figure 4C the HFD feeding determined an over-expression of this enzyme while both treatments showed a significant reduction of this protein in hepatic tissue. Moreover, we showed an increase in SOCS3 protein expression in hepatic tissues from HFD rats, that was significantly inhibited by butyrate and FBA (P<0.05, Figure 4 D) confirming the restoration of insulin signaling.

**Figure 5:**
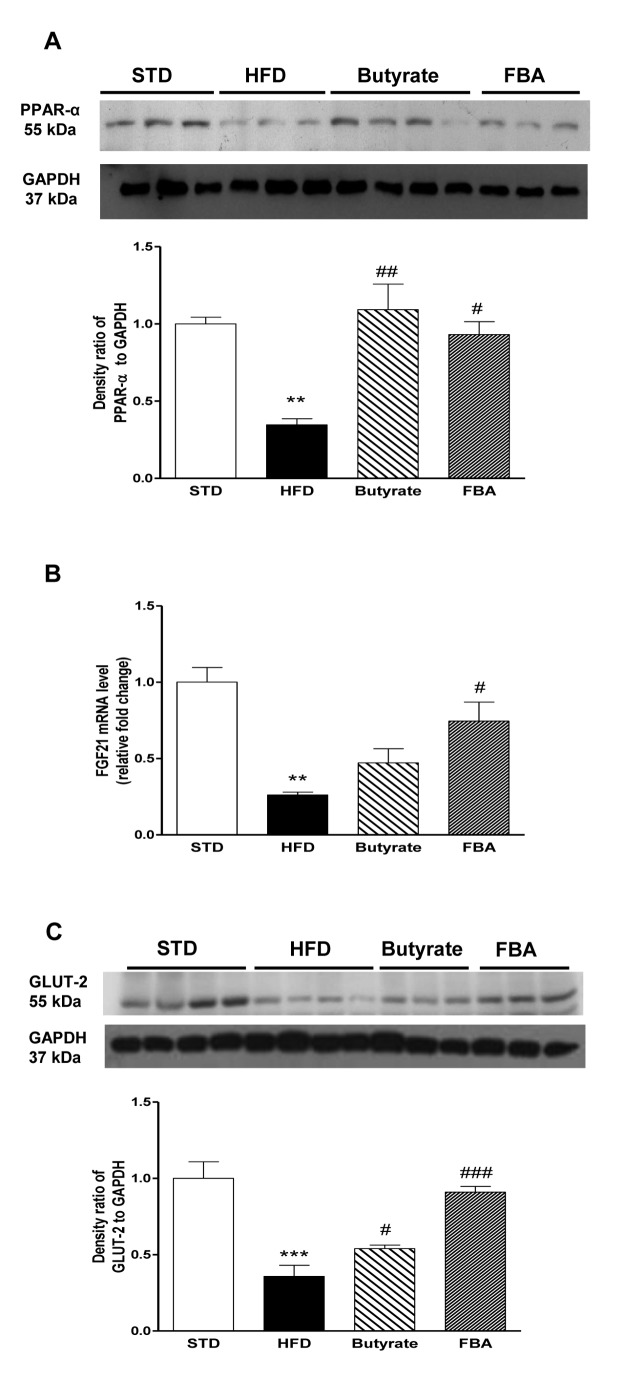
Effect of butyrate and FBA on metabolic impairment and insulin resistance in liver from HFD fed rats. Panel A shows a Western blot analysis of PPAR-α expression (n=6 each group). FGF21 mRNA abundance (B) and GLUT-2 protein expression level (C) in the liver extracts were quantified by real-time PCR and Western blot analysis, respectively (n=6 each group). **P<0.01, ***P<0.001 *vs* STD; ^#^ P<0.05, ^# #^ P<0.01, ^# # #^ P<0.001 *vs* HFD.

According with the inflammatory alterations, other modifications demonstrated metabolic impairment and insulin tissue resistance in HFD fed rats. The basal level of PPAR-α, which regulates fatty acid β-oxidation and catabolism, was detected in liver homogenates from STD fed animals. PPAR-α decreased in liver from HFD rats (P<0.01) and restored by butyrate or FBA (P<0.01 and P<0.05, respectively, Figure 5A). Very recently, it has been demonstrated that FGF21, a cytokine/hormone that plays an important role in the regulation of lipid and carbohydrate metabolism, was induced by butyrate [12]. In liver extracts, FGF21 was significantly reduced by HFD and its decreased expression was slightly prevented by butyrate and significantly by FBA (Figure 5B). Moreover, GLUT2, a glucose-sensitive gene in liver cells [16], was markedly reduced by HFD and this effect was significantly prevented by butyrate and FBA (and Figure 5C).

**Figure 6:**
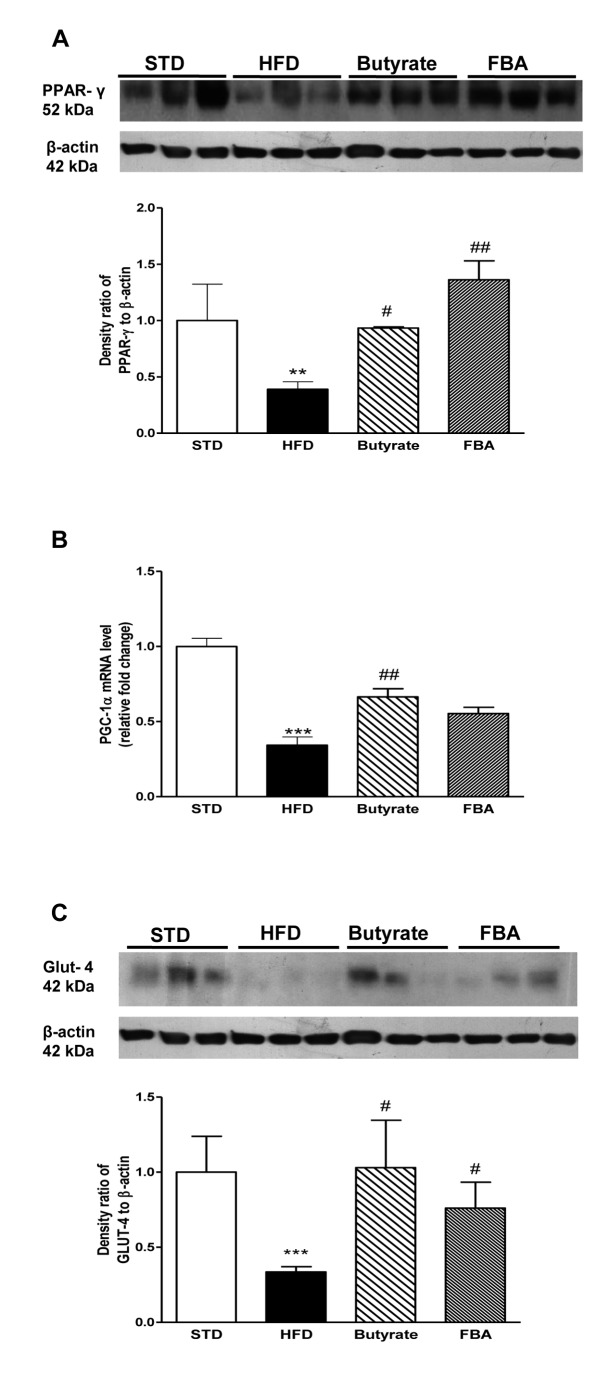
Butyrate and FBA are able to modulate metabolic proteins in adipose tissue. Representative Western blot of PPAR-γ (A) is shown (n=5 each group). Panel B shows real-time PCR results from PGC-1α mRNA levels (relative expression to STD) in adipose tissue (n=6 each group). (C) Western blot analysis of GLUT-4 protein expression in adipose tissue extracts is shown (n=5 each group). **P<0.01 and ***P<0.001 *vs* STD; ^#^ P<0.05, ^# #^ P<0.01 *vs* HFD.

It is well known that adipose tissue, behind its reserve and secretory role, represents an important insulin-sensitive target tissue contributing to glucose homeostasis. Here, the expression of PPAR-γ, a ligand-dependent transcription factor, was lower in HFD compared with STD group, whereas both treatments significantly increased it (Figure 6A). Consistently, the PPAR-γ transcription coactivator, (PGC)-1α, was significantly reduced in HFD group, while both treatments prevented this effect (Figure 6B). It is well known that this coactivator controls energy metabolism interacting with several transcription factors, including PPAR-α, and its reduction is associated with mitochondrial dysfunction, reduction in fatty acid oxidation and insulin resistance [17].

Moreover, in adipose tissue butyrate and FBA were also able to restore GLUT-4 protein expression, that was deeply reduced by HFD (P<0.05, respectively, Figure 6C).

## Discussion

Metabolic and anti-inflammatory effects of butyrate and its derivative, phenylalanine-butyramide (FBA), were examined in this study in a HFD-induced rat model of hepatic steatosis and insulin resistance. Sodium butyrate shows considerable drawbacks: it has fairly strong hygroscopicity and butirric nauseating smell, associated to a poor compliance related to extremely unpleasant taste and epigastric discomfort after oral assumption. On the other hand, the conjugate of butyrate to phenylalanine, FBA, has allowed to obtain a molecule with chemical-physical characteristics suitable for an easier oral administration compared to butyrate.

The most important observation is that both treatments prevented the impairment of glucose homeostasis and the development of insulin resistance. In our model there are two major hallmarks of insulin resistance: hepatic inflammatory process and alteration of glucose tolerance related to an impairment of insulin signaling. The improvement in insulin sensitivity may be, at least in part, a consequence of anti-inflammatory effects of these compounds in this nutritional model. Insulin controls whole body glucose homeostasis with several mechanisms, such as the promotion of glucose disposal in sensitive target tissues, such as liver and fat.

Our study shows that both treatments are able to prevent liver inflammation and damage, steatosis, the onset of IR, and imbalance of TLRs pattern in the early stage of NAFLD. In particular, we showed a reduction in hepatic lipid accumulation mainly significant in FBA treated rats, together with a reduction of the inflammatory infiltrates. The evaluation of the fibrotic process revealed no appreciable modification in HFD fed rats, and consequently no modification by both butyric treatments (data not shown), even if TGF-β, an early pro-fibrogenic marker, was up-regulated in liver from rats on HFD, and markedly reduced by both treatments. TGF-β is considered the most powerful mediator of hepatic stellate cell activation and kupffer cells are a main source of TGF-β in the liver, promoting, collectively to inflammatory cytokines and oxidative stress, later fibrosis [18].

Moreover, both treatments normalize the hepatic markers of steatosis, and preserve glucose tolerance, reducing fasting glucose and modulating HOMA-IR. It is now clear that TNF-α and IL-6 represent crucial effectors of IR, that link liver inflammatory process to hormonal and metabolic alterations [19,20]. In our experimental model, both compounds reduce the above reported cytokines in parallel with a lower expression of PTP1B and SOCS3 inhibitory proteins, suggesting two molecular mechanisms of insulin sensitization. In accordance, recently it has been highlighted how the prototypic phoshotyrosine-specific phoshatase PTP1B dephosphorylates the insulin receptor and downstream IRS-1/2 proteins. Its inhibition enhances insulin signaling and attenuates insulin resistance both in conventional and non conventional insulin-responsive tissues [21], indicating PTP1B as a target for the development of novel therapeutics for diabetes and obesity. On the other hand, SOCS family, including SOCS3, associates with the insulin receptor and inhibits its signaling through ubiquitin-induced degradation of IRS-1. In particular, the induction of SOCS3 in liver may be an important mechanism of IL-6-mediated insulin resistance [19]. Moreover, both treatments reduce the activation of NF-κB pathway induced by HFD, reducing IκBα degradation and inhibiting p50, with a subsequent reduction not only in cytokine transcription (TNFα, IL-6 and IL-1β), but also in inflammatory enzymes (COX-2 and iNOS). Furthermore, we found that HFD increases macrophage infiltration markers, MCP-1 and F4/80, and both butyrate treatments are able to reduce these parameters, in particular reducing F4/80-positive macrophage, indicative of M1state, associated with inflammation and tissue injury [22].

Here, we demonstrate that both treatments reduce inflammation and its mediators inhibiting NF-κB activation arguably through the down-regulation of TLR pattern in the liver, which are involved in bacterial sensing and are crucial for “liver tolerance”. We demonstrate that FBA and, to a lesser extent, butyrate are able to limit the increased transcription and expression of TLRs induced by HFD. The increased expression of hepatic TLRs confirms a greater exposure of the liver to ligands for these receptors deriving from the intestine (i.e. pathogen-associated molecular patterns or PAMPs and endogenous damage-associated molecular patters or DAMPs), including the widely expressed nucleus protein HMGB1. Recently, it has been hypothesized that FFA-related HMGB1 release mediates the activation of TLR4 signaling in hepatocytes and plays an essential part in the early stage of NAFLD induced by HFD [15]. Here, hepatic HMGB1 transcription was strongly up-regulated by HFD feeding while both butyrate, and in particular, FBA determined a significant reduction of its levels. In parallel, they also normalized the expression of TLR4 and its co-receptor CD14 in liver. Accordingly, we evidenced the same profile of activity of butyric treatments on TLR2 mRNA. Consistently with our data, Ehses et al [23] have reported that TLR2 deficient mice are protected from IR and β cell dysfunction induced by HFD, linking TLR2 to the increased dietary lipid and the alteration of glucose homeostasis. Finally, FBA significantly inhibits the HFD-related increase in TLR9 synthesis. Intracellular TLR9 activates innate immune defenses against viral and bacterial infection and plays a role in the pathogenesis of NASH [24].

We previously demonstrated that HFD feeding is associated with the reduction of PPAR-α expression in liver [25] and the administration of a PPAR-α agonist or probiotics restores PPAR-α and improves hepatic steatosis [25,26]. Here, we evidence that butyrate and FBA are also able to do this.

Our data confirm recent *in vitro* and *in vivo* findings that identify butyrate as a new inducer of FGF21 [12]. In that study, butyrate injection increased FGF21 serum concentration and protein levels in the liver of obese mice through activation of PPAR-α which was dependent on HDAC3 inhibition by butyrate.

Despite the differences in the two experimental models (6 weeks vs 20 weeks of diet, an sub-chronic treatment vs an acute one), in our experiments, butyrate and its derivative are still able to restore hormone levels in the early stage of NAFLD, in liver of non obese rats assuming the involvement of HDAC3 inhibition. FGF21 is a metabolic hormone predominantly produced by the liver, but also expressed in adipocytes and pancreas, where it regulates glucose and lipid metabolism through pleiotropic actions [27]. We studied PPAR-α and PPAR-γ expression in tissues where they are more abundant, liver and adipose tissue, respectively [28]. PPAR-γ promotes fatty acid uptake and increases insulin sensitivity by up-regulating GLUT-4, an insulin dependent glucose transporter in adipose tissue and striated muscle [29] and attenuating the induction of SOCS3 [30]. In the current report, we provide evidence that butyrate and FBA not only prevent, in adipose tissue, the HFD-induced reduction of PPAR-γ but also positively modulate PPAR-γ-coactivator PGC-1α [31]. This transcription coactivator controls energy metabolism, interacting with several transcription factors, including PPAR-α and PPAR-δ, that regulate gene transcription for mitochondrial biogenesis and respiration [32]. In fact, the reduction in PGC-1α function is associated with mitochondrial dysfunction, reduction in fatty acid oxidation, and risk for IR or type 2 diabetes [33,34]. Dietary intervention of PGC-1α activity holds promise in the prevention and treatment of metabolic syndrome [11,35]. Our data support the concept that the stimulation of PGC-1α activity may be a molecular mechanism of butyrate activity, in agreement with previous data demonstrating that the inhibition of histone deacetylases and activation of AMPK may contribute to the PGC-1α regulation [11].

Consistently, butyrate and FBA are able to normalize the facilitative hexose transporter, GLUT-2 and insulin-stimulated glucose transport GLUT-4 expression in liver and adipose tissue, respectively, supporting systemic effects of both compounds. Our data are in agreement with previous findings demonstrating that butyrate up-regulates GLUT2 mRNA abundance in other cell types, such as Caco_2_-BBe monolayers, by activating specific regions within the human GLUT2 promoter [36]. GLUT-2 is located in the plasma membrane of hepatocytes and pancreatic beta cells, in contrast with GLUT-1 and GLUT-3, has a low affinity for glucose. Their high Km (15-20mM) allows for glucose sensing; rate of glucose entry is proportional to blood glucose levels. GLUT4 transporters are insulin sensitive, and are found mainly in muscle and adipose tissue. As muscle is a principle storage site for glucose and adipose tissue for triglyceride (into which glucose can be converted for storage), GLUT4 is important in post-prandial uptake of excess glucose from the bloodstream or in other conditions of over-nutrition (i.e. HFD). Moreover, recent findings have reported an improvement of liver glycogen storage by acute butyrate supply that was explained by the competition between butyrate and glucose oxidation, and by a likely reduced glycogenolysis from the newly synthesized glycogen [37].

To our knowledge, our work is the first to propose concomitant biochemical mechanisms in the liver and adipose tissue to better understand how butyrate and its derivative may regulate glucose metabolism and improve insulin sensitivity. Our results show a protective effect of butyrate to limit early molecular events underlying IR linked to steatosis, suggesting a potential clinical utility as innovative, preventive and therapeutic strategy for NAFLD. In fact, these treatments prevent the transition from steatosis toward steatohepatitis, dampening the onset of several hits responsible for the shift and the progression of the disease. Since FBA does not have the characteristic odor of rancid butyrate, this derivative may represent a viable therapeutic alternative to butyrate, favoring a better compliance and a greater effectiveness.

## Materials and Methods

### Ethics Statement

This study was carried out in strict accordance with the Institutional Guidelines and complied with the Italian D.L. no. 116 of January 27, 1992 of Ministero della Salute and associated guidelines in the European Communities Council Directive of November 24, 1986 (86/609/ECC). All animal procedures reported herein were approved by the Institutional Committee on the Ethics of Animal Experiments (CSV) of the University of Naples “Federico II” and by the Ministero della Salute under protocol no. 2008-0099793. Prior to sample collection, animals were euthanized by an intraperitoneal injection of a cocktail of ketamine/xylazine, followed by cervical dislocation to minimize pain. All efforts were made to minimize animal suffering.

### Drugs and reagents

Standard and high-fat diet (HFD) were purchased from Laboratorio Dottori Piccioni (Gessate, Milan, Italy). Standard diet had 15% fat, 22% proteins, and 63% carbohydrates, while HFD had 58% of energy derived from fat, 18% from protein, and 24% from carbohydrates. The composition of high fat diet has been previously described [38]. Standard and high fat diets contained 4.06 kcal/g and 5.56 kcal/g, respectively.

Sodium butyrate was purchased by Sigma-Aldrich (Milan Italy) and phenylalanine-butyramide (FBA; Italian patent RM2008A000214; April 21, 2008) was provided by Prof. Calignano. FBA is present in a solid, poorly hygroscopic, easily weighable form, stable to acids and alkalis and capable of releasing butyric acid at small and large bowel level in a constant manner over time. This product has demonstrated a toxicological profile comparable to that of butyrate; it shows physicochemical characteristics distinctly more suitable for extensive clinical use than those of butyrate. A particular aspect of FBA is that it does not present the unpleasant odour of butyrate and is practically tasteless, thus making possible to overcome the main limitation to the use of butyrate in the therapeutic field, namely its very poor palatability. Moreover, the solubility of FBA in water is satisfactory in that it produces clear solutions up to the concentration of 0.1 M and suspensions for higher concentrations.

### Synthesis and characterization of butyric acid derivative FBA

Briefly, 0.01M of phenylalanine carboximide and 0.01M butyroyl chloride were dissolved in 50 ml of chloroform and the resulting mixture were left to react at room temperature for 24h. The mixture, evaporated in vacuo, yields a solid white-coloured residue, that was washed with a 1% sodium bicarbonate solution. The aqueous bicarbonate solution was extracted twice with an equal volume of ethyl acetate to recover an additional fraction of the mixture of derivatives. To isolate the single components, the mixture was treated and processed chromatographically on a silica gel column, using dichloromethane as eluent. The compound was re-crystallised with a mixture of chloroform/n-hexane 1:1 v: v, obtaining a final yield equal to or greater than 50%.

### Animals and treatments

After weaning, young male Sprague-Dawley rats (average body weight 113.0±2.2 g), purchased from Harlan, Italy, were randomly divided into four groups (at least 6 animals for each group) as follows: 1) a control group receiving STD and vehicle *per os* by gavage; 2) HFD-fed group receiving vehicle; 3) HFD-fed group treated by gavage with sodium butyrate (HFD+butyrate, 20 mg/kg/die) or 4) with N-(1-carbamoyl-2-phenyl-ethyl) butyramide (HFD+FBA 42.5 mg/Kg/die, the equimolecular dose of butyrate). The treatments started together with the HFD and continued for 6 weeks.

We used a nutritional model of IR in non-genetically modified animals [39] that after 6 weeks, induced the early events of NAFLD due to fat overnutrition in young animals, excluding age and gender influences.

Blood sample was collected by cardiac puncture and serum obtained. Liver and white adipose tissue were excised and immediately frozen.

### Blood biochemistry

Alanine amino transferase (ALT), aspartate amino transferase (AST), total cholesterol, low density lipoprotein (LDL) and triglycerides (TGL) were measured by standard automated procedures, according to manufacturer’s protocols (Dade Behring Inc., Newark, DE). Fasting insulin concentrations were measured by rat insulin radioimmunoassay kit (Millipore Corporation, Billerica, MA, USA). Blood glucose concentrations were measured using a glucometer (One Touch UltraSmart; Lifescan, Milpitas, CA). Blood NEFA were determined as previously described (Itaya and Ui, 1965).

### Histological Analysis of Liver Tissue and triglycerides content

Liver sections were stained with hematoxylin and eosin or Oil Red O. Steatosis was graded on a scale of 0 (absence of steatosis), 1 (mild), 2 (moderate) and 3 (extensive). Liver triglycerides were determined as previously described [40].

### Oral Glucose Tolerance Test (OGTT) and Insulin Resistance Assessment

Five weeks after the beginning of the experiment, all rats were fasted for 18 h and then underwent a glucose tolerance. Glucose was orally administered (2 g/kg body weight). Blood samples were collected sequentially from the tail vein before, and 30, 60, 90, and 120 min after the glucose load. The area under the glucose concentration versus time curve (AUC) was calculated from time zero, as the integrated and cumulative measure of glycemia up to 120 min for all animals. To compare the course of the glucose concentration among groups, statistical analysis of AUC mean values was performed using the GraphPad Prism software (GraphPad Software, Inc., San Diego, CA).

As index of insulin resistance, HOMA (homeostasis model assessment) was also calculated, using the formula [HOMA = fasting glucose (mmol/L) × fasting insulin (μU/ml)/22.5].

### Western blotting

Liver and white adipose tissues were homogenized and total protein lysates were subjected to SDS-PAGE. Blots were probed with anti-suppressor of cytokine signaling 3 (SOCS3, Santa Cruz Biotechnology, Inc., Santa Cruz, CA, USA), or anti-protein tyrosine phosphatase 1B (PTP1B, Santa Cruz Biotechnology), or anti-COX-2 (Cayman Chemical, Ann Arbor, MI), or anti-iNOS (BD Trusduction, Franklin Lakes, NJ, USA), or anti-peroxisome proliferator-activated receptor α (PPAR-α, Santa Cruz Biotechnology), or anti-PPAR-γ (Novus Biologicals, Littleton, CO, USA), or anti-glucose transporter-4 (GLUT-4, Santa Cruz Biotechnology) or anti-glucose transporter-2 (GLUT-2, Millipore Corporation, Billerica, MA, USA). To evaluate nuclear factor-κB (NF-κB) activation, IκB-α (Santa Cruz Biotechnology) and NF-κB p50 (Santa Cruz Biotechnology) were measured in liver cytosolic or nuclear extracts, respectively. Western blot for glyceraldehyde-3-phosphate dehydrogenase (GAPDH) or β-actin (in cell lysates, Sigma-Aldrich, Milan, Italy) or lamin A (in nuclei lysates, Chemicon. Int, Temecula, CA, USA) was performed to ensure equal sample loading.

### Real-time semi-quantitative PCR

Total RNA, isolated from liver and adipose tissue, was extracted using TRIzol Reagent (Invitrogen Biotechnologies), according to the manufacturer’s instructions. cDNA was synthesized using a reverse transcription kit (Maxima First Strand cDNA Synthesized Kit, Fermentas, Ontario, Canada) from 2 µg total RNA. PCRs were performed with a Bio-Rad CFX96 Connect Real-time PCR System instrument and software (Bio-Rad Laboratories). The primer sequences are reported in Table 2. The PCR conditions were 10 min at 95°C followed by 40 cycles of two-step PCR denaturation at 95°C for 15 s and annealing extension at 60°C for 60 s. Each sample contained 1-100 ng cDNA in 2X Power SYBRGreen PCR Master Mix (Applied Biosystem) and 200 nmol/l of each primer (Eurofins MWG Operon, Huntsville, AL) in a final volume of 25 µl. The relative amount of each studied mRNA was normalized to GAPDH as housekeeping gene, and the data were analyzed according to the 2^-ΔΔCT^ method.

**Table 2:** Real-Time PCR Primer Sequences.

**Target gene**	**Forward primer (5’→3’)**	**Revere primer (3’→5’)**	**Accession Number**
**CD14**	GTGCTCCTGCCCAGTGAAAGAT	GATCTGTCTGACAACCCTGAGT	AF_087943
**F4/80**	CCCAGCTTATGCCACCTGCA	TCCAGGCCCTGGAACATTGG	NM_001007557.1
**FGF21**	AGATCAGGGAGGATGGAACA	ATCAAAGTGAGGCGATCCATA	NM_130752.1
**GAPDH**	GGCACAGTCAAGGCTGAGAATG	ATGGTGGTGAAGACGCCAGTA	NM_017008 XM_216453
**HMGB-1**	TTGTGCAAACTTGCCGGGAGGA	ACTTCTCCTTCAGCTTGGC	NM_012963.2
**IL-1β**	TCCTCTGTGACTCGTGGGAT	TCAGACAGCACGAGGCATTT	NM_031512
**IL-6**	ACAAGTGGGAGGCTTAATTACACAT	TTGCCATTGCACAACTCTTTTC	NM_012589
**MCP-1**	CCCACTCACCTGCTGCTACT	TCTGGACCCATTCCTTCTTG	NM_031530.1
**PGC1-α**	AACCAGTACAACAATGAGCCTG	AATGAGGGCAATCCGTCTTCA	NM_031347.1
**Pro-collagen type 1**	TCGATTCACCTACAGCACGC	GACTGTCTTGCCCCAAGTTCC	NM_053304.1
**α-SMA**	TGCTGGACTCTGGAGATGG	GATGGTGATCACCTGCCCATC	NM_031004.2
**TGF-β**	GAAGCCATCCGTGGCCAGAT	TGACGTCAAAAGACAGCCACT	NM_021578.2
**TLR2**	GTACGCAGTGAGTGGTGCAAGT	TGGCCGCGTCATTGTTCTC	NM_198769 XM_227315
**TLR4**	CTACCTCGAGTGGGAGGACA	ATGGGTTTTAGGCGCAGAGTT	NM_019178
**TLR9**	ATGGCCTGGTAGACTGCAACT	TTGGCGATCAAGGAAAGGCT	NM_198131
**TNF-α**	CATCTTCTCAAAACTCGAGTGACAA	TGGGAGTAGATAAGGTACAGCCC	NM_012675

### Statistical analysis

All data were presented as mean ± SEM. Statistical analysis was performed by ANOVA test followed by Bonferroni’s test for multiple comparisons. Statistical significance was set at *P*<0.05.

## References

[1] Pryde SE, Duncan SH, Hold GL, Stewart CS, Flint HJ (2002) The microbiology of butyrate formation in the human colon. FEMS Microbiol Lett 217: 133-139. doi:10.1111/j.1574-6968.2002.tb11467.x. PubMed: 12480096. 12480096 10.1111/j.1574-6968.2002.tb11467.x

[2] Roy CC, Kien CL, Bouthillier L, Levy E (2006) Short-chain fatty acids: ready for prime time? Nutr Clin Pract 21: 351–366. doi:10.1177/0115426506021004351. PubMed: 16870803. 16870803 10.1177/0115426506021004351

[3] Ritzaupt A, Wood IS, Hosie Ellis A. KB, Shirazi-Beechey SP (1998) Identification and characterization of a monocarboxylate transporter (MCT1) in pig and human colon: its potential to transport L-lactate as well as butyrate. J Physiol 513: 719-732

[4] Binder HJ, Mehta P (1989) Short-chain fatty acids stimulate active sodium and chloride absorption in vitro in the rat distal colon. Gastroenterology 96: 989-996. PubMed: 2925072. 2925072 10.1016/0016-5085(89)91614-4

[5] Berni Canani R, Terrin G, Cirillo P, Castaldo G, Salvatore F et al. (2004) Butyrate as an effective treatment of congenital chloride diarrhea. Gastroenterology 127: 630-634. doi:10.1053/j.gastro.2004.03.071. PubMed: 15300594. 15300594 10.1053/j.gastro.2004.03.071

[6] Davie JR (2003) Inhibition of histone deacetylase activity by butirate activity. J Nutr 133: 2485S-2493S. PubMed: 12840228. 12840228 10.1093/jn/133.7.2485S

[7] Berni Canani R, Di Costanzo M, Leone L (2012) The epigenetic effects of butyrate: potential therapeutic implications for clinical practice. Clin Epigenetics 4: 4. doi:10.1186/1868-7083-4-4. PubMed: 22414433. 22414433 10.1186/1868-7083-4-4 PMC3312834

[8] Glozak MA, Sengupta N, Zhang X, Seto E (2005) Acetylation and deacetylation of non-histone proteins. Gene 363: 15-23. doi:10.1016/j.gene.2005.09.010. PubMed: 16289629. 16289629 10.1016/j.gene.2005.09.010

[9] Brown AJ, Goldsworthy SM, Barnes AA, Eilert MM, Tcheang L et al. (2003) The Orphan G protein-coupled receptors GPR41 and GPR43 are activated by propionate and other short chain carboxylic acids. J Biol Chem 278: 11312–11319. doi:10.1074/jbc.M211609200. PubMed: 12496283. 12496283 10.1074/jbc.M211609200

[10] Berni Canani R, Costanzo MD, Leone L, Pedata M, Meli R et al. (2011) Potential beneficial effects of butyrate in intestinal and extraintestinal diseases. World J Gastroenterol 17: 1519-1528. doi:10.3748/wjg.v17.i12.1519. PubMed: 21472114. 21472114 10.3748/wjg.v17.i12.1519 PMC3070119

[11] Gao Z, Yin J, Zhang J, Ward RE, Martin RJ et al. (2009) Butyrate improves insulin sensitivity and increases energy expenditure in mice. Diabetes 58: 1509-1517. doi:10.2337/db08-1637. PubMed: 19366864. 19366864 10.2337/db08-1637 PMC2699871

[12] Li H, Gao Z, Zhang J, Ye X, Xu A et al. (2012) Sodium butyrate stimulates expression of fibroblast growth factor 21 in liver by inhibition of histone deacetylase 3. Diabetes 61: 797-806. doi:10.2337/DB12-DualityInfo. PubMed: 22338096. 22338096 10.2337/db11-0846 PMC3314370

[13] Kharitonenkov A, Shiyanova TL, Koester A, Ford AM, Micanovic R et al. (2005) FGF-21 as a novel metabolic regulator. J Clin Invest 115: 1627-1635. doi:10.1172/JCI23606. PubMed: 15902306. 15902306 10.1172/JCI23606 PMC1088017

[14] Shi H, Kokoeva MV, Inouye K, Tzameli I, Yin H et al. (2006) TLR4 links innate immunity and fatty acid-induced insulin resistance. J Clin Invest 116: 3015-3025. doi:10.1172/JCI28898. PubMed: 17053832. 17053832 10.1172/JCI28898 PMC1616196

[15] Li L, Chen L, Hu L, Liu Y, Sun HY et al. (2011) Nuclear factor high-mobility group box1 mediating the activation of Toll-like receptor 4 signaling in hepatocytes in the early stage of nonalcoholic fatty liver disease in mice. Hepatology 54: 1620-1630. doi:10.1002/hep.24552. PubMed: 21809356. 21809356 10.1002/hep.24552

[16] Rencurel F, Waeber G, Antoine B, Rocchiccioli F, Maulard P et al. (1996) Requirement of glucose metabolism for regulation of glucose transporter type 2 (GLUT2) gene expression in liver. Biochem J 314: 903-909. PubMed: 8615787. 8615787 10.1042/bj3140903 PMC1217142

[17] Petersen KF, Befroy D, Dufour S, Dziura J, Ariyan C et al. (2003) Mitochondrial dysfunction in the elderly: possible role in insulin resistance. Science 300: 1140–1142. doi:10.1126/science.1082889. PubMed: 12750520. 12750520 10.1126/science.1082889 PMC3004429

[18] De Minicis S, Svegliati-Baroni G (2011) Fibrogenesis in nonalcoholic steatohepatitis. Expert Rev Gastroenterol Hepatol 5: 179-187. doi:10.1586/egh.11.28. PubMed: 21476913. 21476913 10.1586/egh.11.28

[19] Senn JJ, Klover PJ, Nowak IA, Zimmers TA, Koniaris LG et al. (2003) Suppressor of cytokine signaling-3 (SOCS-3), a potential mediator of interleukin-6-dependent insulin resistance in hepatocytes. J Biol Chem 278: 13740-13746. doi:10.1074/jbc.M210689200. PubMed: 12560330. 12560330 10.1074/jbc.M210689200

[20] Hotamisligil GS, Peraldi P, Budavari A, Ellis R, White MF et al. (1996) IRS-1-mediated inhibition of insulin receptor tyrosine kinase activity in TNF-alpha- and obesity-induced insulin resistance. Science 271: 665-668. doi:10.1126/science.271.5249.665. PubMed: 8571133. 8571133 10.1126/science.271.5249.665

[21] Tiganis T (2013) PTP1B and TCPTP - nonredundant phosphatases in insulin signaling and glucose homeostasis. FEBS J 280: 445-458. doi:10.1111/j.1742-4658.2012.08563.x. PubMed: 22404968. 22404968 10.1111/j.1742-4658.2012.08563.x

[22] Kanda H, Tateya S, Tamori Y, Kotani K, Hiasa K et al. (2006) MCP-1 contributes to macrophage infiltration into adipose tissue, insulin resistance, and hepatic steatosis in obesity. J Clin Invest 116: 1494-1505. doi:10.1172/JCI26498. PubMed: 16691291. 16691291 10.1172/JCI26498 PMC1459069

[23] Ehses JA, Meier DT, Wueest S, Rytka J, Boller S et al. (2010) Toll-like receptor 2-deficient mice are protected from insulin resistance and beta cell dysfunction induced by a high-fat diet. Diabetologia 53: 1795-1806. doi:10.1007/s00125-010-1747-3. PubMed: 20407745. 20407745 10.1007/s00125-010-1747-3

[24] Miura K, Kodama Y, Inokuchi S, Schnabl B, Aoyama T et al. (2010) Toll-like receptor 9 promotes steatohepatitis by induction of interleukin-1beta in mice. Gastroenterology 139: 323-334. doi:10.1053/j.gastro.2010.03.052. PubMed: 20347818. 20347818 10.1053/j.gastro.2010.03.052 PMC4631262

[25] Esposito E, Iacono A, Bianco G, Autore G, Cuzzocrea S et al. (2009) Probiotics reduce the inflammatory response induced by a high-fat diet in the liver of young rats. J Nutr 139: 905-911. doi:10.3945/jn.108.101808. PubMed: 19321579. 19321579 10.3945/jn.108.101808

[26] Harano Y, Yasui K, Toyama T, Nakajima T, Mitsuyoshi H et al. (2006) Fenofibrate, a peroxisome proliferator-activated receptor alpha agonist, reduces hepatic steatosis and lipid peroxidation in fatty liver Shionogi mice with hereditary fatty liver. Liver Int 26: 613-620. doi:10.1111/j.1478-3231.2006.01265.x. PubMed: 16762007. 16762007 10.1111/j.1478-3231.2006.01265.x

[27] Woo YC, Xu A, Wang Y, Lam KS (2013) Fibroblast Growth Factor 21 as an emerging metabolic regulator: clinical perspectives. Clin Endocrinol (Oxf) 78: 489-496. doi:10.1111/cen.12095. PubMed: 23134073. 23134073 10.1111/cen.12095

[28] Wang YX (2010) PPARs: diverse regulators in energy metabolism and metabolic diseases. Cell Res 20: 124-137. doi:10.1038/cr.2010.13. PubMed: 20101262. 20101262 10.1038/cr.2010.13 PMC4084607

[29] Gurnell M (2003) PPARgamma and metabolism: insights from the study of human genetic variants. Clin Endocrinol 59: 267-277. doi:10.1046/j.1365-2265.2003.01767.x. 10.1046/j.1365-2265.2003.01767.x 12919147

[30] Chatterjee PK (2010) Hepatic inflammation and insulin resistance in pre-diabetes - further evidence for the beneficial actions of PPAR-gamma agonists and a role for SOCS-3 modulation. Br J Pharmacol 160: 1889-1891. doi:10.1111/j.1476-5381.2010.00739.x. PubMed: 20649587. 20649587 10.1111/j.1476-5381.2010.00739.x PMC2958634

[31] Lagouge M, Argmann C, Gerhart-Hines Z, Meziane H, Lerin C et al. (2006) Resveratrol improves mitochondrial function and protects against metabolic disease by activating SIRT1 and PGC-1alpha. Cell 127: 1109–1122. doi:10.1016/j.cell.2006.11.013. PubMed: 17112576. 17112576 10.1016/j.cell.2006.11.013

[32] Lin J, Handschin C, Spiegelman BM (2005) Metabolic control through the PGC-1 family of transcription coactivators. Cell Metab 1: 361–370. doi:10.1016/j.cmet.2005.05.004. PubMed: 16054085. 16054085 10.1016/j.cmet.2005.05.004

[33] Patti ME, Butte AJ, Crunkhorn S, Cusi K, Berria R et al. (2003) Coordinated reduction of genes of oxidative metabolism in humans with insulin resistance and diabetes: potential role of PGC1 and NRF1. Proc Natl Acad Sci U S A 100: 8466–8471. doi:10.1073/pnas.1032913100. PubMed: 12832613. 12832613 10.1073/pnas.1032913100 PMC166252

[34] Mootha VK, Lindgren CM, Eriksson KF, Subramanian A, Sihag S et al. (2003) PGC-1alpha-responsive genes involved in oxidative phosphorylation are coordinately downregulated in human diabetes. Nat Genet 34: 267–273. doi:10.1038/ng1180. PubMed: 12808457. 12808457 10.1038/ng1180

[35] Baur JA, Pearson KJ, Price NL, Jamieson HA, Lerin C et al. (2006) Resveratrol improves health and survival of mice on a high-calorie diet. Nature 444: 337–342. doi:10.1038/nature05354. PubMed: 17086191. 17086191 10.1038/nature05354 PMC4990206

[36] Mangian HF, Tappenden KA (2009) Butyrate increases GLUT2 mRNA abundance by initiating transcription in Caco_2_-BBe cells. J Parenter Enteral Nutr 33: 607-617. doi:10.1177/0148607109336599. PubMed: 19892901. 10.1177/0148607109336599 19892901

[37] Beauvieux MC, Roumes H, Robert N, Gin H, Rigalleau V et al. (2008) Butyrate ingestion improves hepatic glycogen storage in the re-fed rat. BMC Physiol 8: 19. doi:10.1186/1472-6793-8-19. PubMed: 18847460. 18847460 10.1186/1472-6793-8-19 PMC2569010

[38] Surwit RS, Feinglos MN, Rodin J, Sutherland A, Petro AE et al. (1995) Differential effects of fat and sucrose on the development of obesity and diabetes in C57BL/6J and A/J mice. Metabolism 44: 645–651. doi:10.1016/0026-0495(95)90123-X. PubMed: 7752914. 7752914 10.1016/0026-0495(95)90123-x

[39] Svegliati-Baroni G, Candelaresi C, Saccomanno S, Ferretti G, Bachetti T et al. (2006) A model of insulin resistance and nonalcoholic steatohepatitis in rats: role of peroxisome proliferator-activated receptor-alpha and n-3 polyunsaturated fatty acid treatment on liver injury. Am J Pathol 169: 846-860. doi:10.2353/ajpath.2006.050953. PubMed: 16936261. 16936261 10.2353/ajpath.2006.050953 PMC1698833

[40] Sardesai VM, Manning JA (1967) The determination of tiglycerides in plasma and tissues. Clin Chem 14: 156-161.

